# Supplementary material for: Repeated Social Defeat Exaggerates Fibrin-Rich Clot Formation by Enhancing Neutrophil Extracellular Trap Formation via Platelet–Neutrophil Interactions
Source: Cells. 2021 Nov 28;10(12):3344. doi: 10.3390/cells10123344 (PMC8699805; doi:10.3390/cells10123344)
Supplement: Supplementary file 1 [file cells-10-03344-s001.zip › cells-1470344-supplementary.pdf]

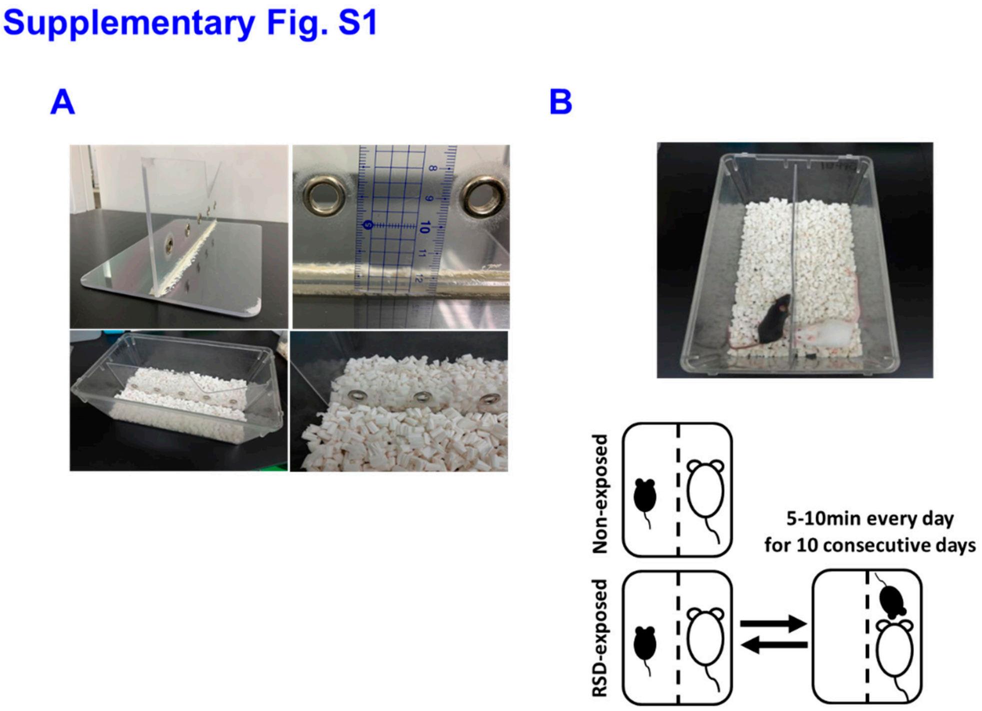

Figure S1. Apparatuses for the exposure to social stress. (A) Photographs of perforated partition. **(B)** Photograph of housed mice without physical contact. RSD, repeated social defeat.

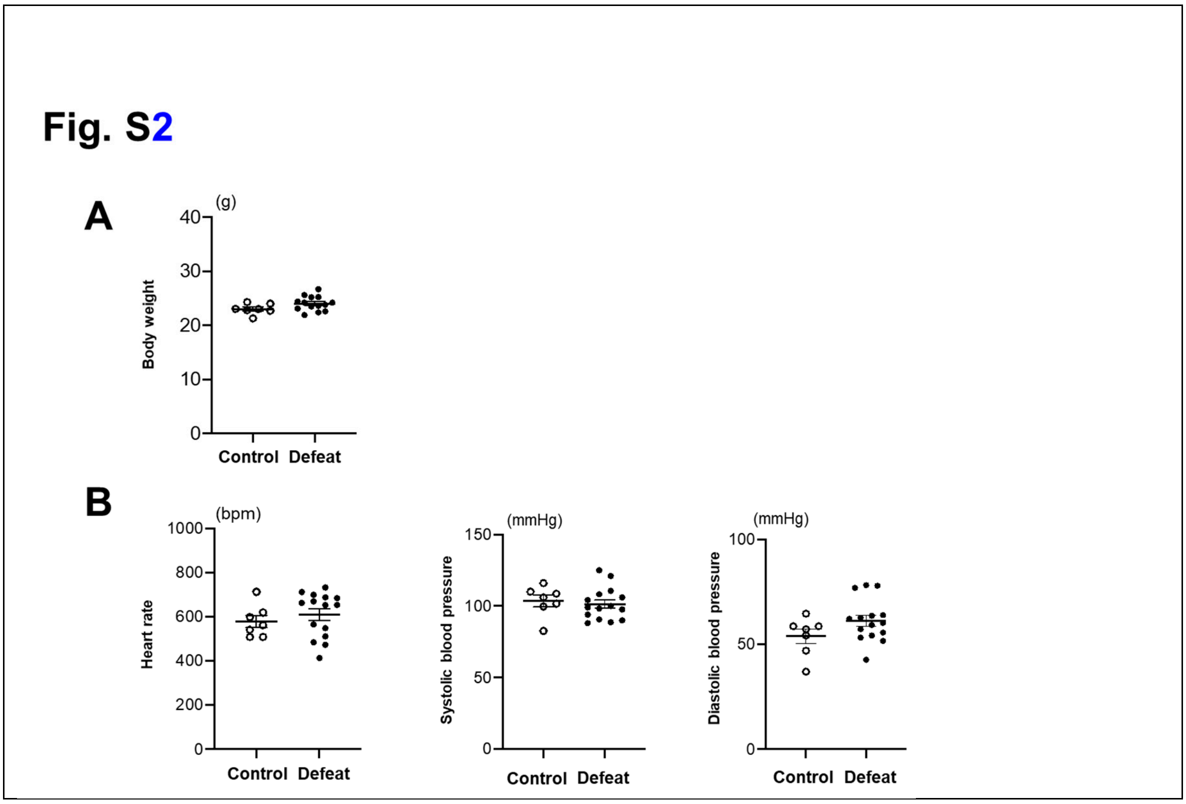

Fig. S2

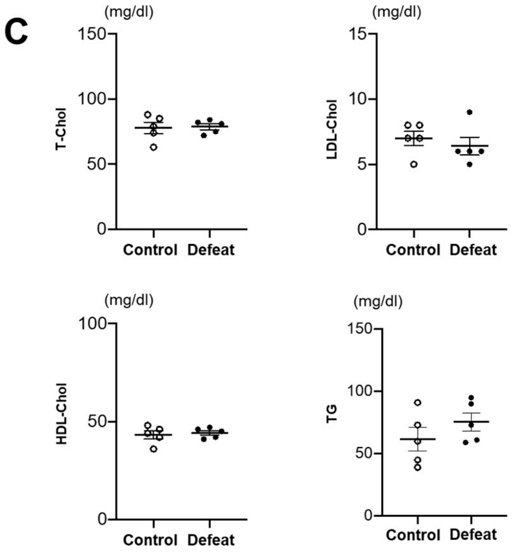

Figure S2. Body weight, hemodynamic parameters, and lipid profiles before $FeCl_3$ application are comparable between the two groups. (A and B) Body weight, blood pressure and heart rate are similar between the two groups. Values represent mean ± standard error of mean (SEM) for seven control and 15 defeated mice. **(C)** Lipid profiles show no significant difference between the two groups. Values represent mean ± standard error of mean (SEM) for five control and five defeated mice. T-Chol: Total cholesterol. LDL-Chol: low-density lipoprotein cholesterol. HDL-Chol: high-density lipoprotein cholesterol. TG: triglyceride.

Fig. S3

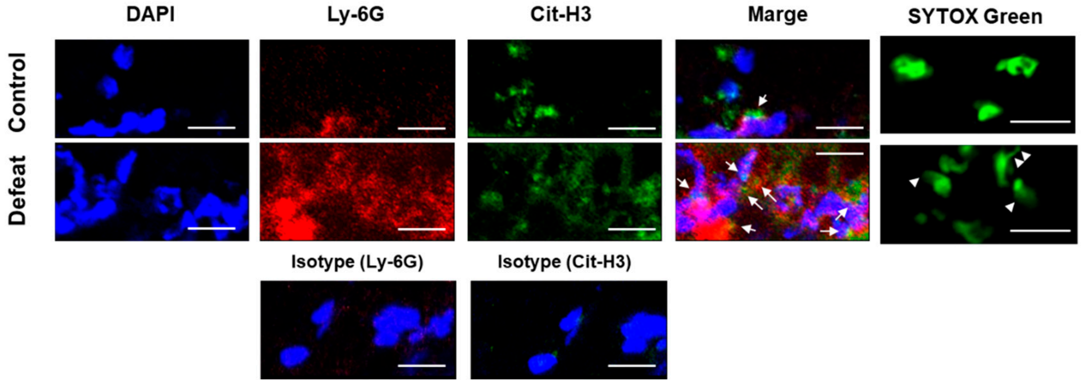

Figure S3. Immunofluorescent imaging of NET formation in thrombi. The arrows indicate Ly-6G/Cit-H3 double-positive cells, while the arrow heads indicate extracellular DNA. Scale bar = 10 μm. Cit-H3, citrullinated histone H3.

Fig. S4

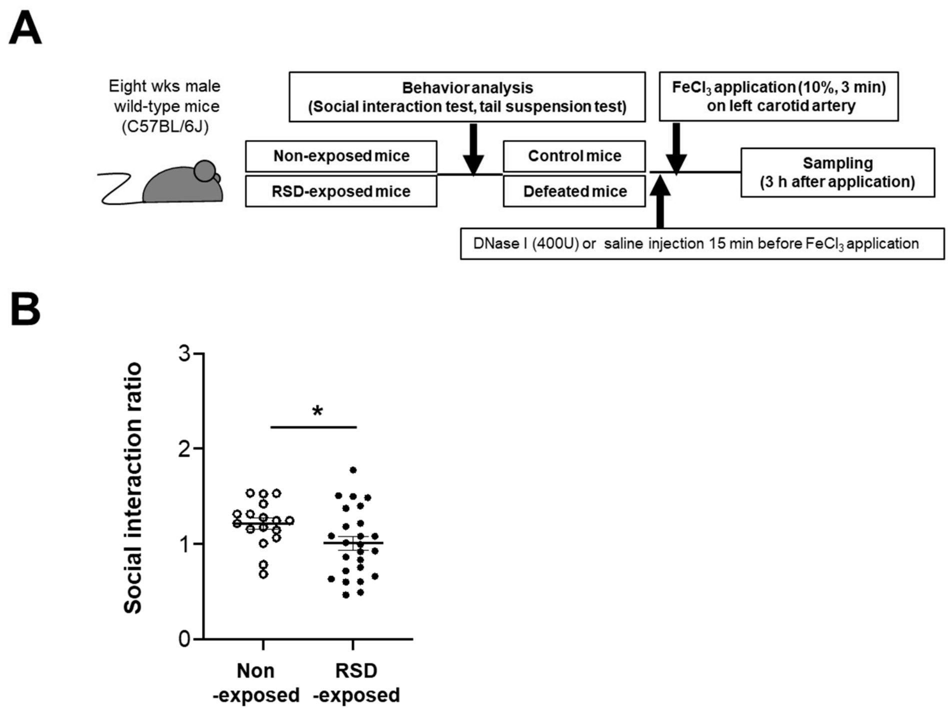

Figure S4. Experimental protocol of DNase I treatment. (A) Timeline of experimental protocol using DNase I. (B) Social interaction test. Values represent mean ± standard error of mean (SEM) for 17 non-exposed and 25 RSD-exposed mice before $FeCl_3$ application. ****p* **< 0.05 vs. non-exposed mice; Student's t-test. RSD, repeated social defeat.**

Fig. S5

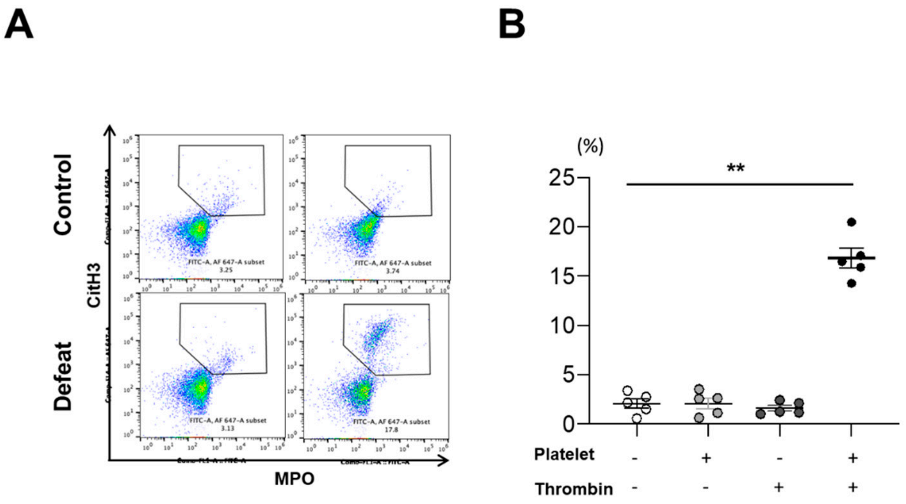

Figure S5. In vitro NET formation using mature BM neutrophils and thrombin-induced activated platelets. (A) Representative flow cytometry data showing Cit-H3- and MPO-positive cells in mature

BM neutrophils from group-housed wild-type mice. **(B)** Values represent mean ± standard error of mean (SEM) for five in each group. ** $p < 0.01$ vs. Platelet (-) Thrombin (-); one-way ANOVA with the Tukey–Kramer post hoc test.

Fig. S6

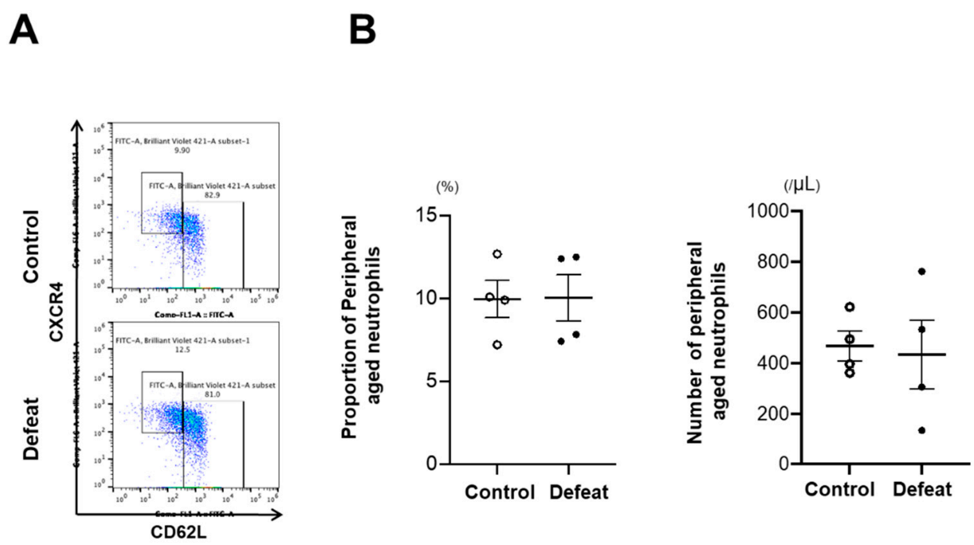

Figure S6. Repeated social defeat does not affect the fraction and number of aged neutrophils before $FeCl_3$ application. (A) Representative flow cytometry data showing $CD62L^{low}CXCR4^{high}$ cells in aged PB neutrophils from control and defeated mice. **(B)** Values represent mean ± standard error of mean (SEM) for four control and four defeated mice.
